# Added Clinical Benefit of Voiding Cystourethrography Combined With Standard-of-Care Retrograde Urethrography for Adult Male Urethral Strictures and Urethral Injuries: A Pictorial Review

**DOI:** 10.7759/cureus.111528

**Published:** 2026-06-26

**Authors:** Konstantinos Douroumis, Konstantinos Kotrotsios, Panagiotis Makris, Panagiotis Levis, Napoleon Moulavasilis, Konstandinos Stravodimos, Ioannis Adamakis

**Affiliations:** 1 First Urology Department, National and Kapodistrian University of Athens, Athens, GRC

**Keywords:** retrograde urethrography, urethral injury, urethral stenosis, urethral stricture disease, voiding cystourethrography

## Abstract

Precise imaging constitutes the cornerstone of effective urethral stricture and injury management. Although retrograde urethrography (RUG) is widely used as the investigation of choice to assess the anterior urethra, it comes with several limitations. In some cases, voiding cystourethrography (VCUG) can provide complementary anatomical and functional information that can be highly influential on clinical decision-making. More specifically, VCUG allows accurate visualization of the urethra proximal to the stricture, as well as a more reliable assessment of the stricture’s length in virtually obliterative strictures and stenoses, and gaps in case of pelvic fracture injury. Also, it can introduce unique data, such as bladder neck integrity, prostatic urethra patency, and dynamic voiding patterns. This information can often impact the therapeutic plan, including the choice between endoscopic and open approach, timing of repair, and continence prognosis. In this pictorial review, by providing examples from our clinical practice and describing our technique, we aim to illustrate the imaging spectrum of urethral strictures and injuries on VCUG, highlighting its additional diagnostic input and discussing common interpretive pitfalls.

## Introduction and background

Accurate radioscopic visualization of the entire urethral lumen is a critical factor in preoperative decision-making in reconstructive urology. Abnormalities of the urethral lumen mainly consist of either lumen narrowing or trauma-related lesions. Narrowing of the urethral lumen in men, when affecting the anterior urethra, refers to a process of fibrosis and cicatrization of the urethral tissue and surrounding spongiosus tissue, which is defined as a urethral stricture, while, when the narrowing affects the posterior urethra, where no spongiosus tissue is present, the term urethral stenosis is preferred [1]. Urethral trauma, mainly sub-categorized into iatrogenic and non-iatrogenic trauma, is characterized by disruption of the physiological continuity of the urethral lumen, and further management is dependent on the location and severity of injury [2].

When abnormalities of the urethral lumen are suspected, due to either traumatic urethral lumen rupture or non-traumatic lumen narrowing, retrograde urethrography (RUG) is the gold standard imaging modality for the initial assessment of the urethral lumen [3,4]. RUG allows for evaluation of the diseased urethral lumen, providing information about the location and length of lesions as well as any concomitant anomalies such as false urethral routes and/or diverticula [5]. Visualization of the urethral lumen, as enabled by RUG, allows for accurate urethral stricture diagnosis with 91% sensitivity and 72% specificity [6]. Regarding urethral trauma, although several classifications according to the degree of contrast medium extravasation and bladder filling during RUG have been proposed, differentiation between complete and incomplete rupture is not always distinct, and thus, only localization of the traumatic lesion in the anterior or posterior urethra can be reliably performed [7,8]. Interpretation of RUG should be performed by the treating urologist who can estimate stricture length and location with better accuracy compared to independent radiologists, with significant inter-observer interpretation of findings being observed, even among urologists [9,10]. Special considerations regarding the interpretation of RUG findings in urethral trauma should include assessment of competence and anatomy of the bladder neck, as well as concomitant fractures or injuries that will cause significant deviations from standard surgical planes during surgical repairs [11].

Voiding cystourethrography (VCUG), combined with standard-of-care RUG, enables visualization of the urethral lumen proximal to the bladder, otherwise not feasible in cases of nearly obliterative strictures, long-length stenoses, including the external sphincter, and ruptures of the urethral lumen commonly associated with pelvic fracture urethral injuries (PFUIs) [12,13]. A combination of those imaging modalities provides clinically significant information about bladder neck integrity and contractility, prostatic urethra patency, and bladder filling and voiding patterns that will allow for optimal surgical planning. Currently, only the European Association of Urology (EAU) implements VCUG as standard of care in guideline recommendations, only in cases of patients with nearly obliterative strictures, stenosis, and pelvic fracture urethral injuries [14]. Herein, we present a pictorial review highlighting the added clinical benefit of VCUG in addition to standard-of-care RUG in patients presenting with urethral strictures, stenoses, and urethral trauma.

## Review

Materials and methods

This pictorial review was based on a unique collection of urethral radiographic studies performed at our institution between July 2020 and March 2026 as part of standard preoperative evaluation. Eligible for inclusion were adult male patients with available RUG and VCUG images of sufficient quality demonstrating underlying urethral pathology, who were planned to undergo reconstructive intervention due to urethral stricture, stenosis, or trauma. Patients with early phase urethral trauma, pediatric patients, and patients without sufficient imaging studies to assess either the retrograde or voiding phase were excluded. Since this pictorial review is based on original images from our clinical practice, no formal literature search was performed to identify relevant imaging studies. All urethral visualization studies were retrieved from the institutional imaging archive and were reviewed by two urologists experienced in reconstructive surgery. Due to the educational purpose of this pictorial review, cases indicating the insufficiency of RUG alone, the added clinical benefit of VCUG, and the key anatomical and functional findings capable of altering reconstructive planning were selected. As this study was conducted based on a retrospective review of de-identified imaging studies, formal ethical approval was waived according to institutional policy.

VCUG Technique

The patient is placed in an oblique position at an angle between 35° and 45° with the lower leg flexed and the upper leg extended. A syringe with iodine contrast, diluted in normal saline, is prepared. For the RUG, a Foley catheter (maximum: 14 Fr) is used, and the contrast medium is injected through it. The Foley catheter can be placed with its tip in the fossa navicularis and the balloon inflated with a small amount of distilled water (2 cc), or with the assistance of a clamp device, if it is available. These maneuvers aim to help in applying gentle traction, which facilitates more accurate visualization. In cases where a meatal stricture is present, a 16 Gauge grey flexible intravenous catheter or a 6 Fr ureteric catheter is required. In these cases, it is helpful to wrap a compress under the glans penis to facilitate traction and avoid irradiation of the examiner’s hands [15].

Moderate traction is applied to elongate the urethra, and the contrast medium is instilled gently to avoid extravasation of contrast and subsequent urethral bleeding. The distention of the anterior urethra and the bladder filling are recorded. In case bladder filling is not feasible retrogradely, a suprapubic catheter is placed to enable voiding films to be taken. Adequate bladder filling (250-300 mL) is essential to secure bladder neck opening and the effective voiding phase. Subsequently, the patient is placed in orthostatic and oblique position at a 35°-45° angle, and continuous or intermittent fluoroscopy is performed to visualize the opening of the bladder neck, the passage of the contrast through the membranous and the prostatic urethra, and the distal urethral segment [16].

Since part of the urethra is located deep within the pelvis and the perineum, combined with the innate limitation of fluoroscopy to depict three-dimensional structures onto a two-dimensional image, addressing common pitfalls is important to eliminate artifacts [17]. Given that both position and penile traction can affect the measured length of the stricture, standardized positioning protocols and controlled penile traction should be developed and implemented to reduce variability. Correct positioning can be indicated by a downward-oriented obturator foramen [18]. Also, it should be mentioned that forceful injection of the iodine contrast can lead to a false diagnosis of urethral strictures due to reflex contraction of the pelvic muscles [19].

**Figure 1:**
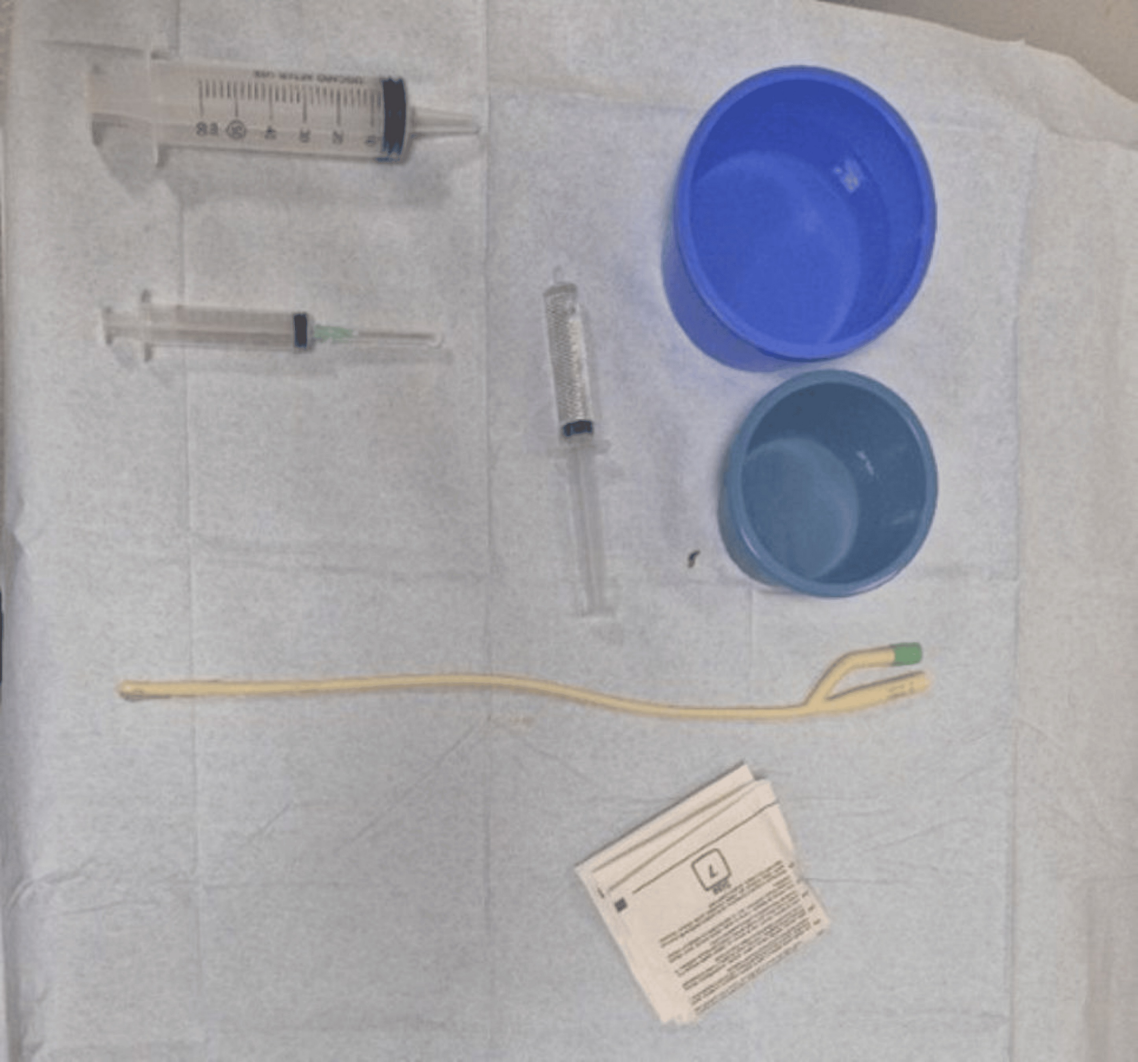
Setup for the urethrogram We use a catheter (maximum: 14 Fr) and 2 cc of water to inflate the balloon in the fossa navicularis. For the contrast medium instillation, a 60 cc syringe is used. Alternatively, in cases with meatal stricture, a 6 Fr ureteric catheter is used. This is an original image from our practice.

Figure 1 presents a setup for the urethrogram.

While there is evidence highlighting the importance of the interpretation of VCUG in conjunction with RUG by a urologist, this is not the case in many settings, where it is performed by residents or radiologists. Less experienced practitioners can misinterpret a contracted external sphincter as a stricture and falsely estimate the normal urethra as a stricture [9].

**Figure 2:**
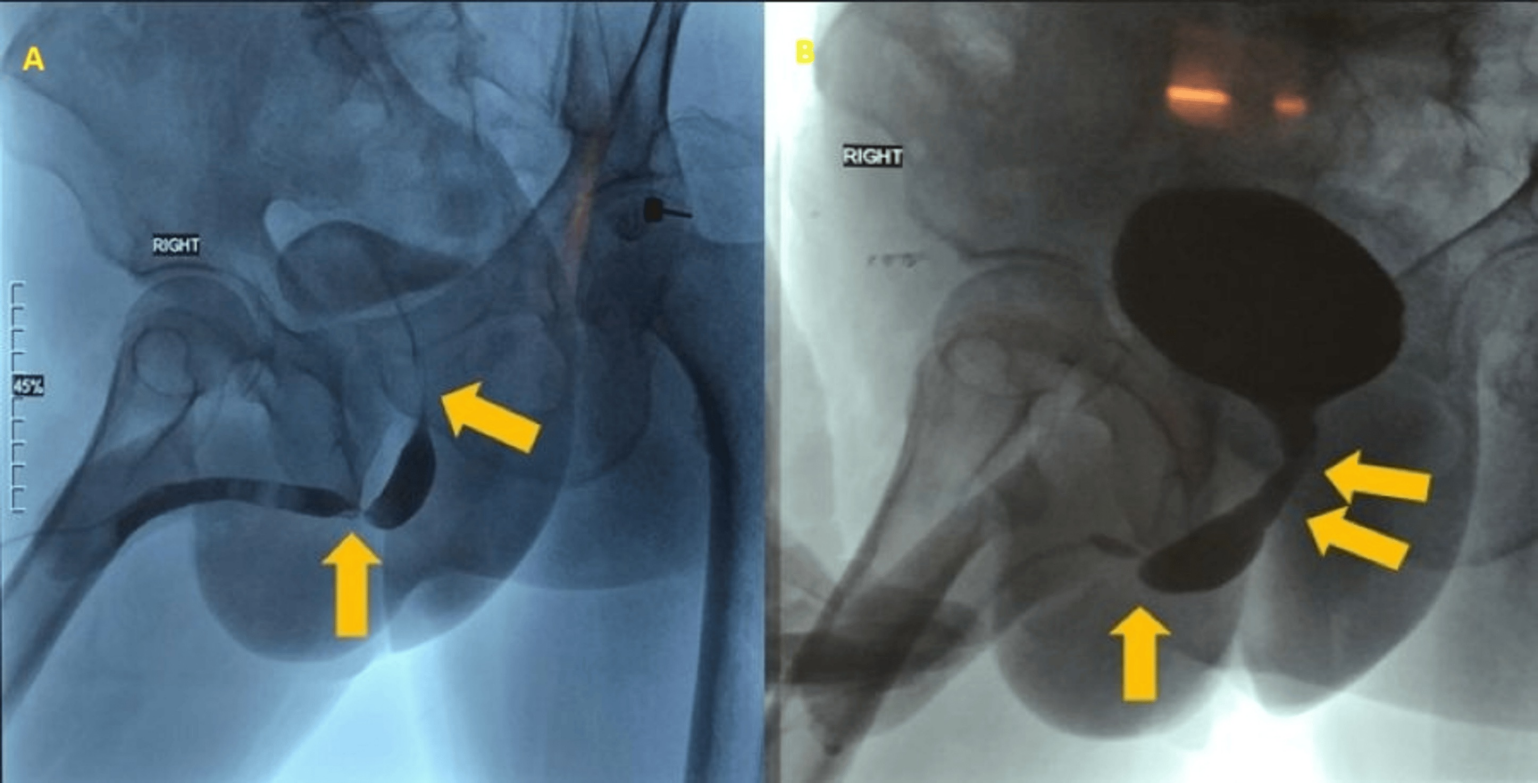
Common pitfall of a closed sphincter A 56-year-old patient was assessed for obstructive micturition. The patient had an IPSS as high as 8, and his prostate volume and post-void residual were 46 cc and 35 cc, respectively. These two images constitute an example of how a closed external sphincter may be mistakenly identified as a urethral stricture; the RUG image is shown in picture A, while the VCUG image is in picture B. Urethroscopy revealed an obstructive prostate, and the patient underwent TURP. The yellow arrows point toward the closed external sphincter in picture A, which could be mistaken for a stricture. However, during voiding depicted in VCUG (image B), the sphincter is open, and the narrowing disappears. RUG: retrograde urethrogram, TURP: transurethral resection of the prostate, VCUG: voiding cystourethrogram, IPSS: International Prostate Symptom Score Consent to publish these images was obtained from the patient

Figure 2 presents a common pitfall of a closed sphincter.

VCUG contraindications

There are no absolute contraindications to performing VCUG in the context of urethral stricture assessment. However, relative contraindications include acute UTI, which should be treated with appropriate antibiotic administration, recent instrumentation, and contrast media allergy [20,21]. Also, special consideration should be taken when performing this procedure in patients with spinal cord injury who are affected above the splanchnic sympathetic outflow tract. Given that bladder filling in these patients can lead to autonomic dysreflexia, it is important to have a catheterization kit ready in case the bladder needs to be emergently drained [22]. In respect of urethral trauma, VCUG is usually carried out three months after the injury to evaluate the site, length, and severity of the injury, as well as the function of the bladder neck, and should be avoided during the acute setting [23].

Additional information provided by VCUG on urethral strictures

Anterior Urethral Strictures

Voiding cystourethrography can introduce additional anatomical, as well as functional, information. Adding VCUG, along with RUG, in the assessment of anterior urethral strictures allows more reliable evaluation of the urethra posteriorly of the stricture, especially in cases of obliterative or nearly obliterative strictures. In fact, the European Association of Urology recommends incorporating VCUG alongside RUG in this context [12,14].

**Figure 3:**
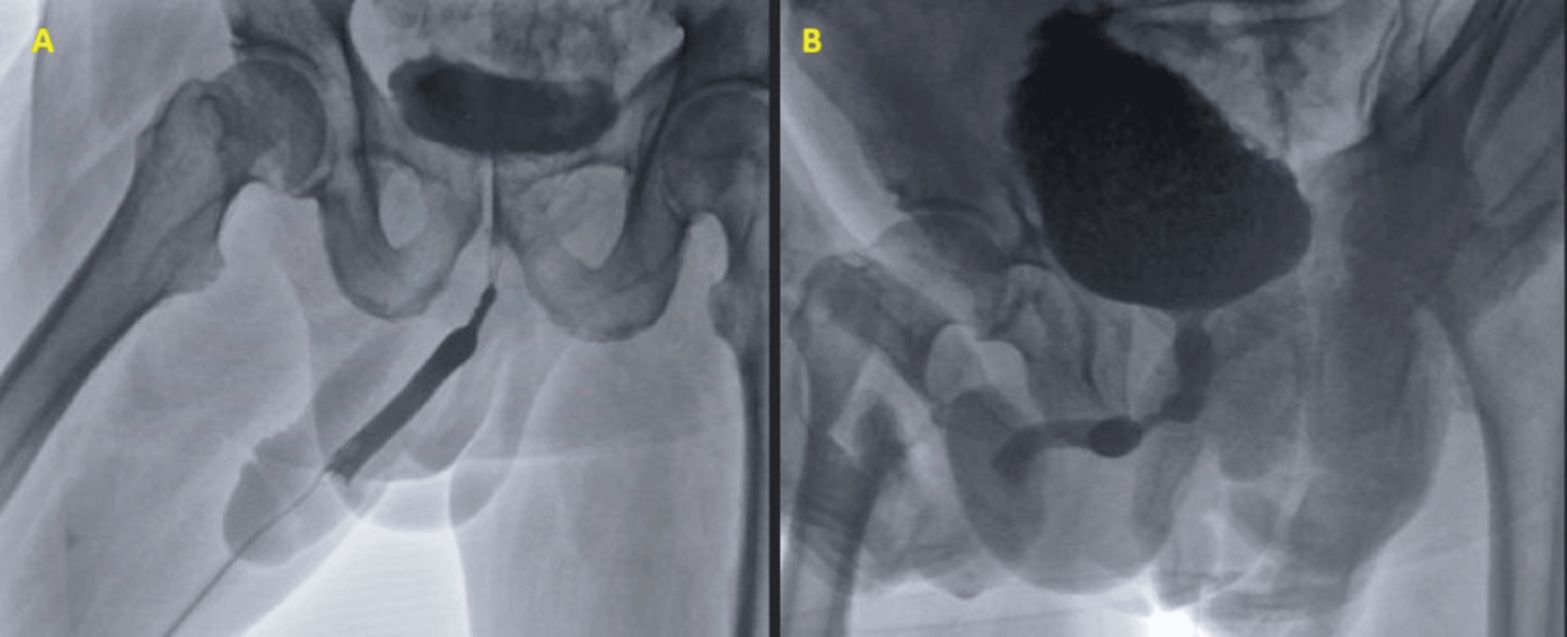
Bulbar urethral stricture This imaging study was performed on a 71-year-old male patient with a history of three DVIUs. In picture A, RUG demonstrates the stricture but is unable to assess its length or the presence of a stricture more proximally. In picture B, VCUG, in this case, provided us with the exact stricture length (2.5 cm). In this case, bladder trabeculation was also revealed with the VCUG. The patient was treated with augmentation urethroplasty. DVIU: direct vision internal urethrotomy, RUG: retrograde urethrogram, VCUG: voiding cystourethrogram Consent to publish these images was obtained from the patient.

Figure 3 illustrates a bulbar urethral stricture.

Additionally, VCUG can offer significant aid in visualization of strictures involving the fossa navicularis by ameliorating the delineation of the stricture’s proximal segment [24]. In these cases, a VCUG can provide more precise information on the stricture’s length, caliber, and exact anatomical location. In this setting, RUG is not able to characterize the strictures, as the contrast column remains continuous and uniformly “thin” along the entire urethral length, while on VCUG, the urethral lumen tapers precisely at the level of the stricture, making the narrowing clearly appreciable. In fact, there are some really rare cases where an obliterative urethral meatus makes catheterization of the urethra impossible, and VCUG through a suprapubic catheter remains the only possible way of evaluating the stricture.

**Figure 4:**
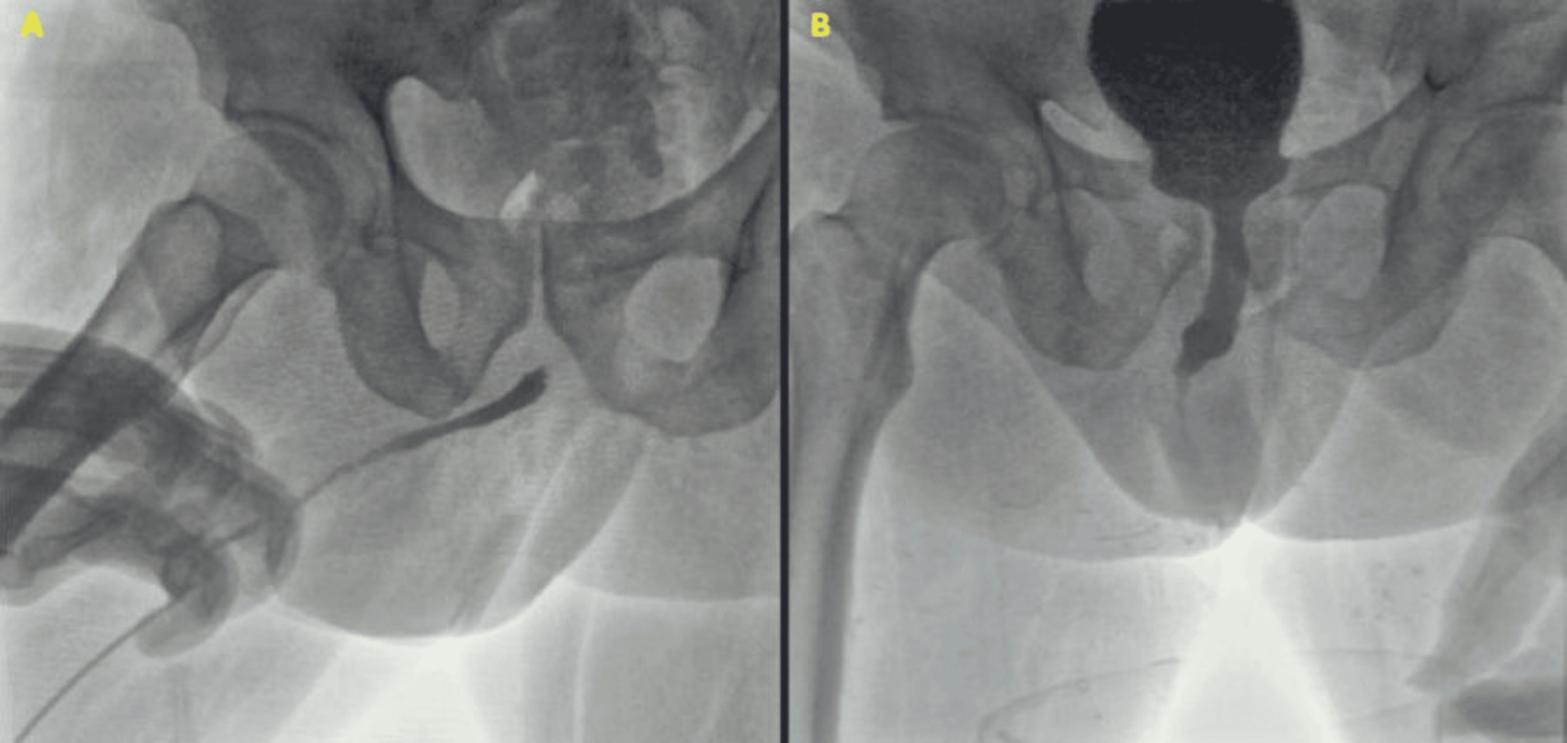
A patient with a meatal stenosis This was a 62-year-old male patient with lichen sclerosus and a history of two DVIUs. For the RUG in picture A, a 6 Fr ureteric catheter was used. The RUG was unable to demonstrate the full extent of the stricture. Incorporation of VCUG, as depicted in picture B, demonstrated a stricture that extends the whole penile urethra. The patient was treated with the “Mini-Kulkarni” approach. DVIU: direct vision internal urethrotomy, RUG: retrograde urethrogram, VCUG: voiding cystourethrogram Consent to publish these images was obtained from the patient.

**Figure 5:**
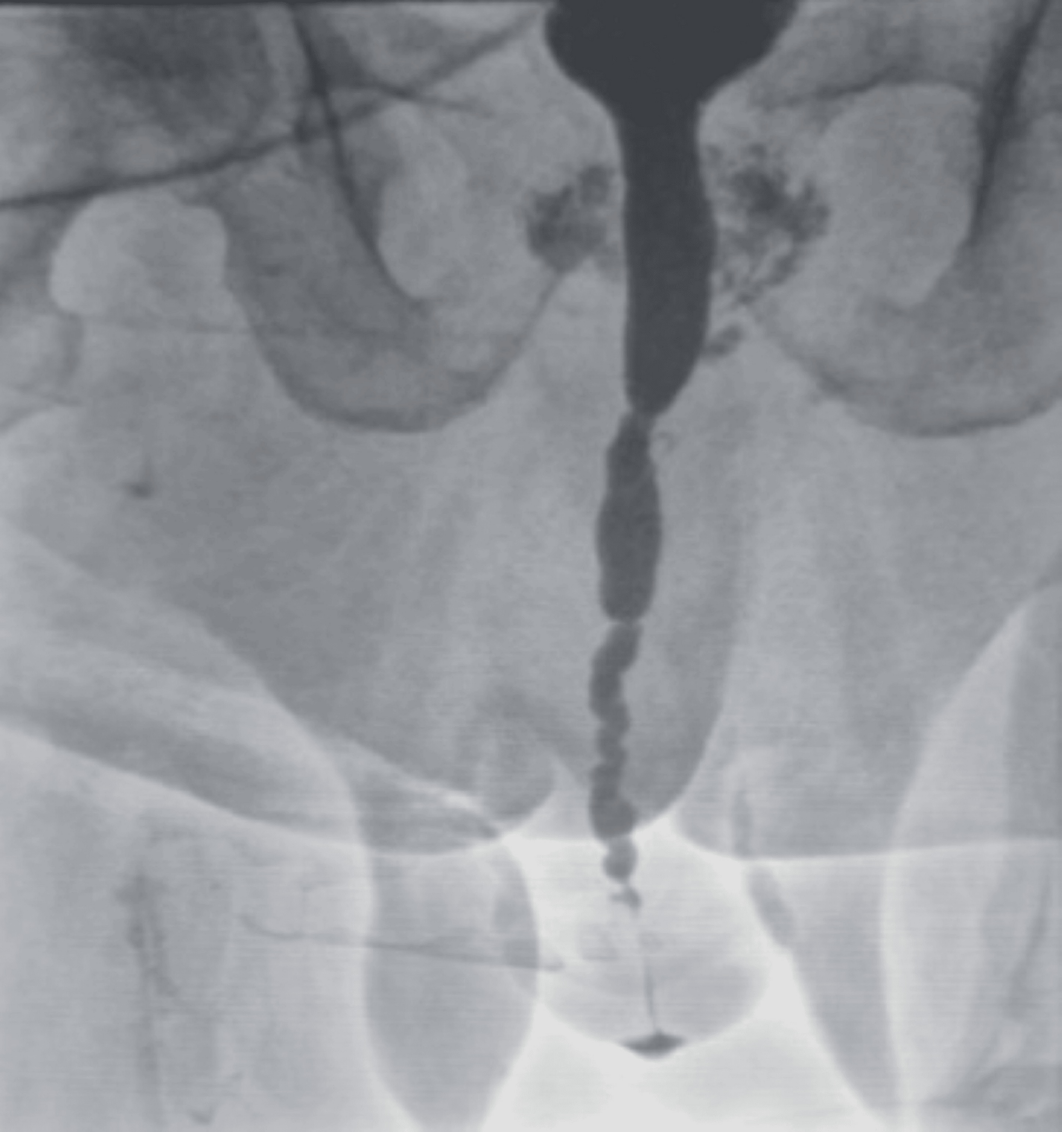
Obliterative meatal stricture This case refers to an 82-year-old male patient with a history of multiple catheterizations. In this case, performing RUG was impossible, and thus, VCUG, through the suprapubic catheter, was the only feasible option, revealing a 3.5-4 cm penile stricture and a 1 cm bulbar stricture. The patient was treated with the “Mini-Kulkarni” approach. RUG: retrograde urethrogram, VCUG: voiding cystourethrogram Consent to publish this image was obtained from the patient.

Figure 4 shows a patient with a meatal stenosis, and Figure 5 presents an obliterative meatal stricture.

Posterior Urethral Stenosis

Normally, the posterior urethra, in patients with adequate external sphincteric function, will be closed in RUG [18]. As such, posterior urethral pathology cannot be identified with RUG. In these cases, VCUG is necessary to characterize prostatic and membranous urethral stenosis. Posterior urethral stenosis is caused mainly after prostatic treatments, such as transurethral resection of the prostate, radical prostatectomy, and radiotherapy [16]. As such, in all patients evaluated with urethrography after prostatic treatment, VCUG must be considered a standard.

**Figure 6:**
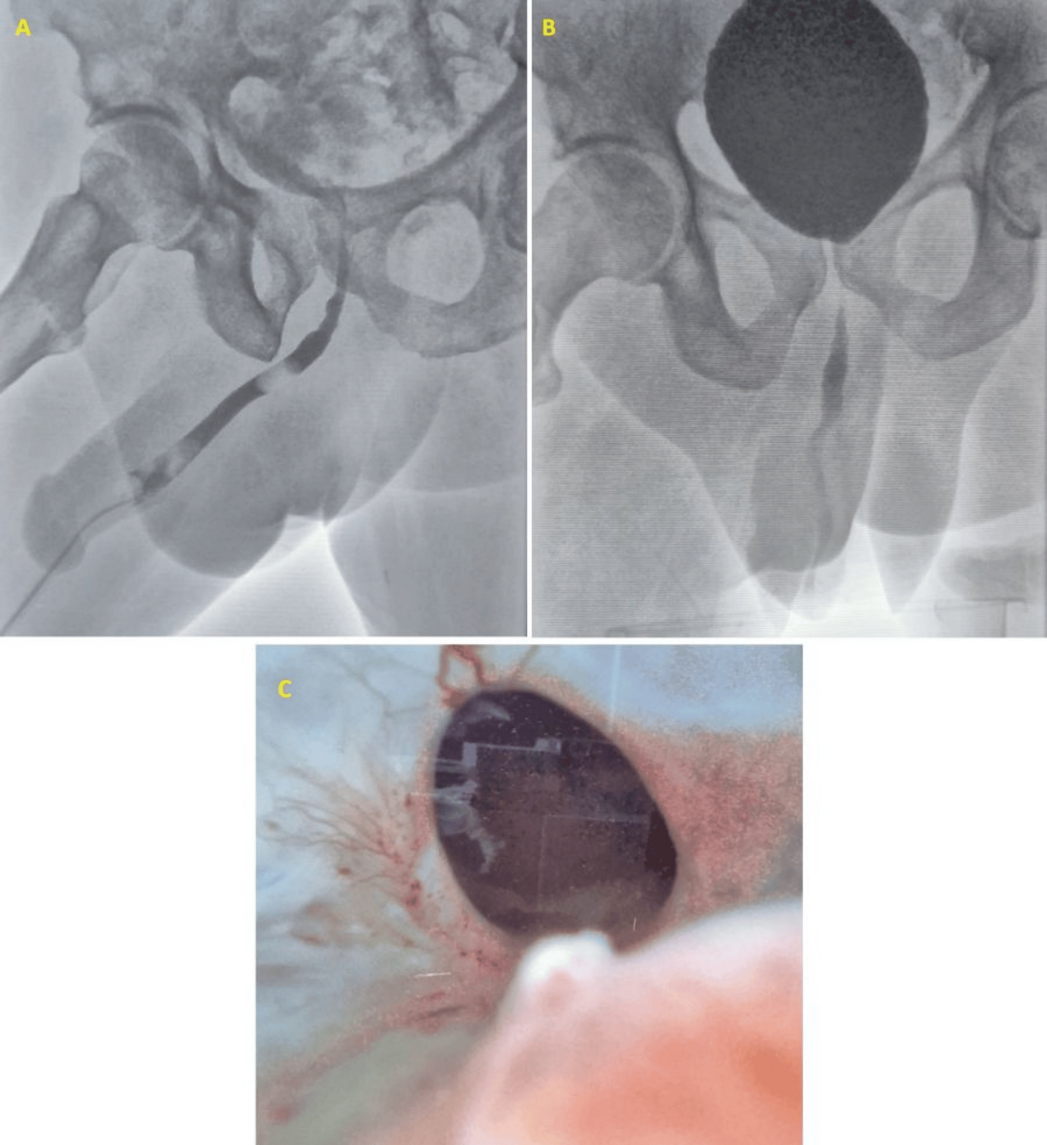
Patient with a bladder neck stenosis In a patient with a history of radical prostatectomy and pelvic radiation, RUG was not diagnostic, as indicated in picture A, because of the closed external sphincter. However, as shown in picture B, VCUG was pathognomonic for a posterior urethral filling defect, with cystoscopy confirming a bladder neck stenosis. RUG: retrograde urethrogram, VCUG: voiding cystourethrogram Consent to publish these images was obtained from the patient.

Figure 6 shows a patient with a bladder neck stenosis.

Bladder Anatomy and Functional Assessment of the Lower Urinary Tract

Reconstructive urology in general, and urethroplasty in particular, is referrals-driven, with a small proportion of urologists performing the majority of urethroplasties [25]. As such, it is not uncommon for the reconstructive urologist to identify previously unrecognized pathologies in patients presenting with urethral strictures.

VCUG has a great role in the detection of emerging pathologies coexisting with urethral strictures. These entities include diverticula, bladder trabeculation, and bladder fistula [18]. Recognizing these preoperatively is of great value, as the surgical plan might have to be modified.

**Figure 7:**
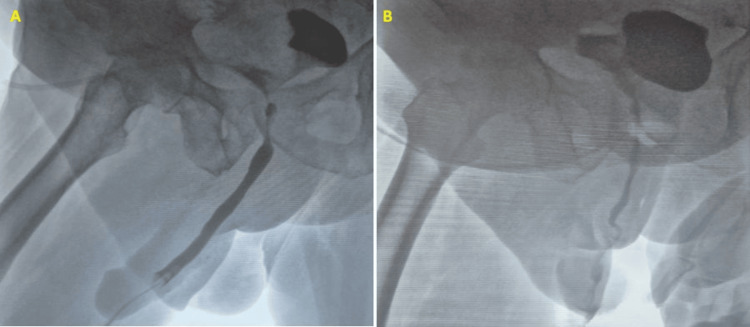
Patient with membranous urethral stenosis and a bladder diverticulum In this case, a 76-year-old male patient, who reported obstructive micturition three months after TURP, was assessed. VCUG, in picture B, helps with both the stenosis, which can be missed in case of a closed sphincter, and in the evaluation of the diverticulum, regarding its size and its neck diameter. In this patient, the diverticulum is visible in the RUG, as shown in picture A, after the bladder has been instilled with an adequate amount of contrast. RUG: retrograde urethrogram, TURP: transurethral resection of the prostate, VCUG: voiding cystourethrogram Consent to publish these images was obtained from the patient.

Figure 7 shows a membranous urethral stenosis and a bladder diverticulum in a patient.

Moreover, VCUG provides a functional evaluation of the micturition by examining the hydrodistension effect of dilatation proximally to the stricture. VCUG enables the identification of functional micturition abnormalities, such as reduced bladder capacity and compliance and vesicourethral reflux, along with identification of neurogenic bladder, with the typical appearance of “Christmas tree’’ [26,27]. It is important to diagnose these entities before a possible urethroplasty, as it can change the treatment plan.

**Figure 8:**
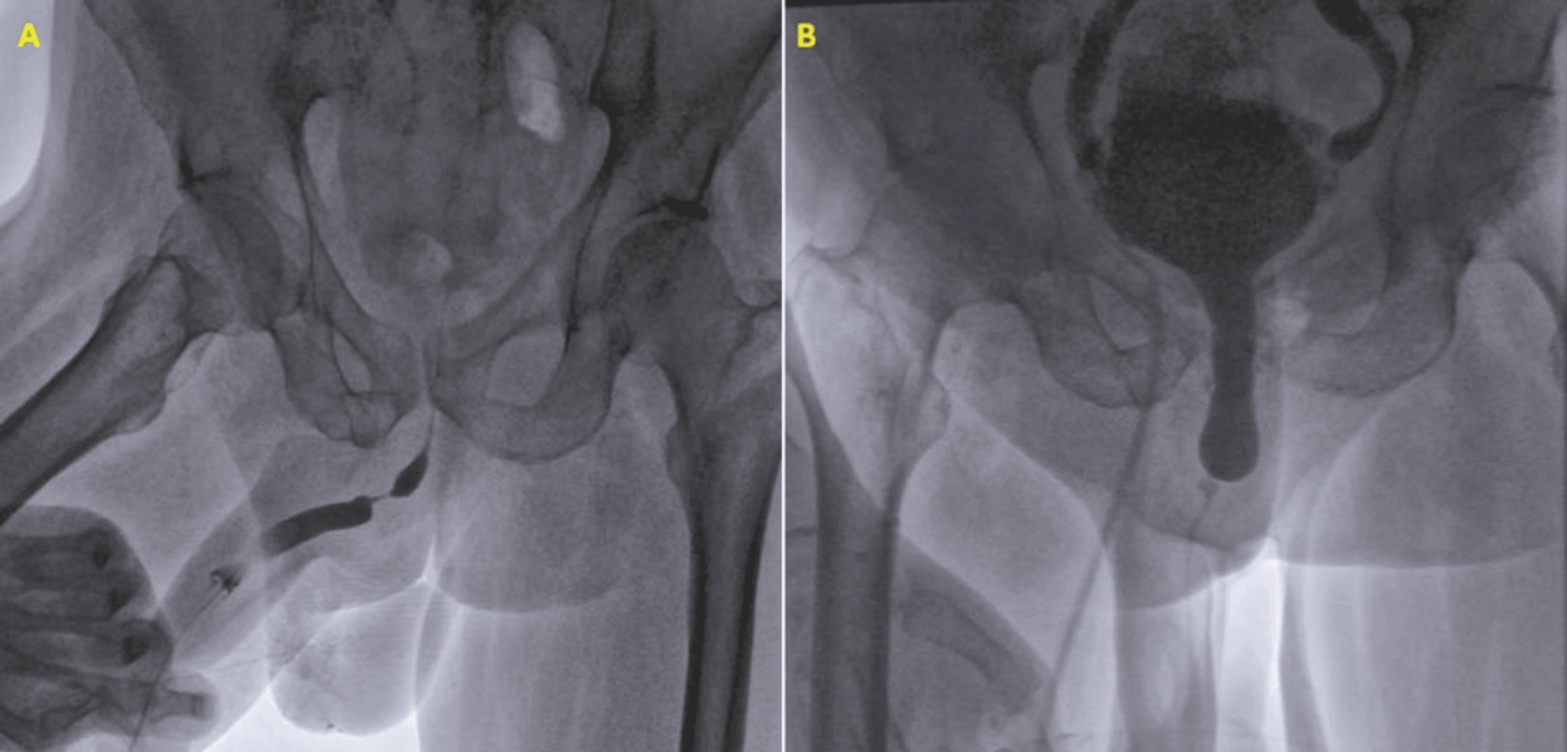
Adult patient evaluated for anterior urethral stricture RUG in picture A revealed a 2.5 cm bulbar stricture, whereas VCUG in picture B revealed bilateral vesicoureteral reflux. This finding prompted further evaluation of the patient. If this patient had been evaluated only with RUG, an augmented urethroplasty would have been performed. This is a typical case of a previously unrecognized pathology, in which VCUG altered the treatment plan. Subsequently, the patient underwent urodynamic investigation. RUG: retrograde urethrogram, VCUG: voiding cystourethrogram Consent to publish these images was obtained from the patient.

Figure 8 shows an anterior urethral stricture in an adult patient.

Additional information provided by VCUG in urethral trauma

RUG is the gold standard modality in the evaluation of male patients with urethral injury, as it can reveal extravasation of the contrast medium outside of the urethra, which is pathognomonic [23]. In this stage, VCUG should be avoided, as instilling a great amount of contrast in the bladder can exacerbate the urethral injury, while it does not add value regarding the diagnosis of the injury.

The standard of care in most of these patients remains the delayed urethroplasty, meaning urethroplasty at least three months after the injury. In this setting, VCUG can provide complementary information in respect of urethral injuries, with its combination with the RUG being the mainstay in urethral injury visualization [2]. This combination allows for the correct estimation of the urethral defect.

**Figure 9:**
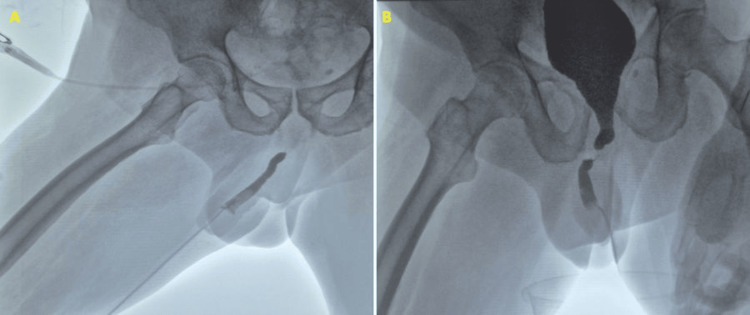
Patient with a complete urethral rupture following urethral injury This 47-year-old male patient suffered from a straddle injury and, in the acute phase, presented with blood at the urethral meatus. The prostate was normally palpable at his anatomical position. One month after the incident, RUG and VCUG were performed. RUG in picture A shows the distal segment of the rupture. The combination of RUG and VCUG in picture B shows the extent of the injury. The patient was treated with urethroplasty. RUG: retrograde urethrogram, VCUG: voiding cystourethrogram Consent to publish these images was obtained from the patient.

Figure 9 shows a complete urethral rupture following urethral injury.

In patients with pelvic fracture urethral injuries (PFUIs), VCUG remains indispensable, in combination with RUG. As these injuries involve the membranous urethra, VCUG should be performed, in combination with RUG, in every stable patient presenting PFUI, to avoid misinterpretation of the true distraction defect length. Additionally, distinction of the level of urethral injury as above or below the lower margin of the pubic symphysis, which is determined through VCUG, based on the location of the posterior stump of the injury, can predict the need for inferior wedge pubectomy and supracrural urethral rerouting [13].

**Figure 10:**
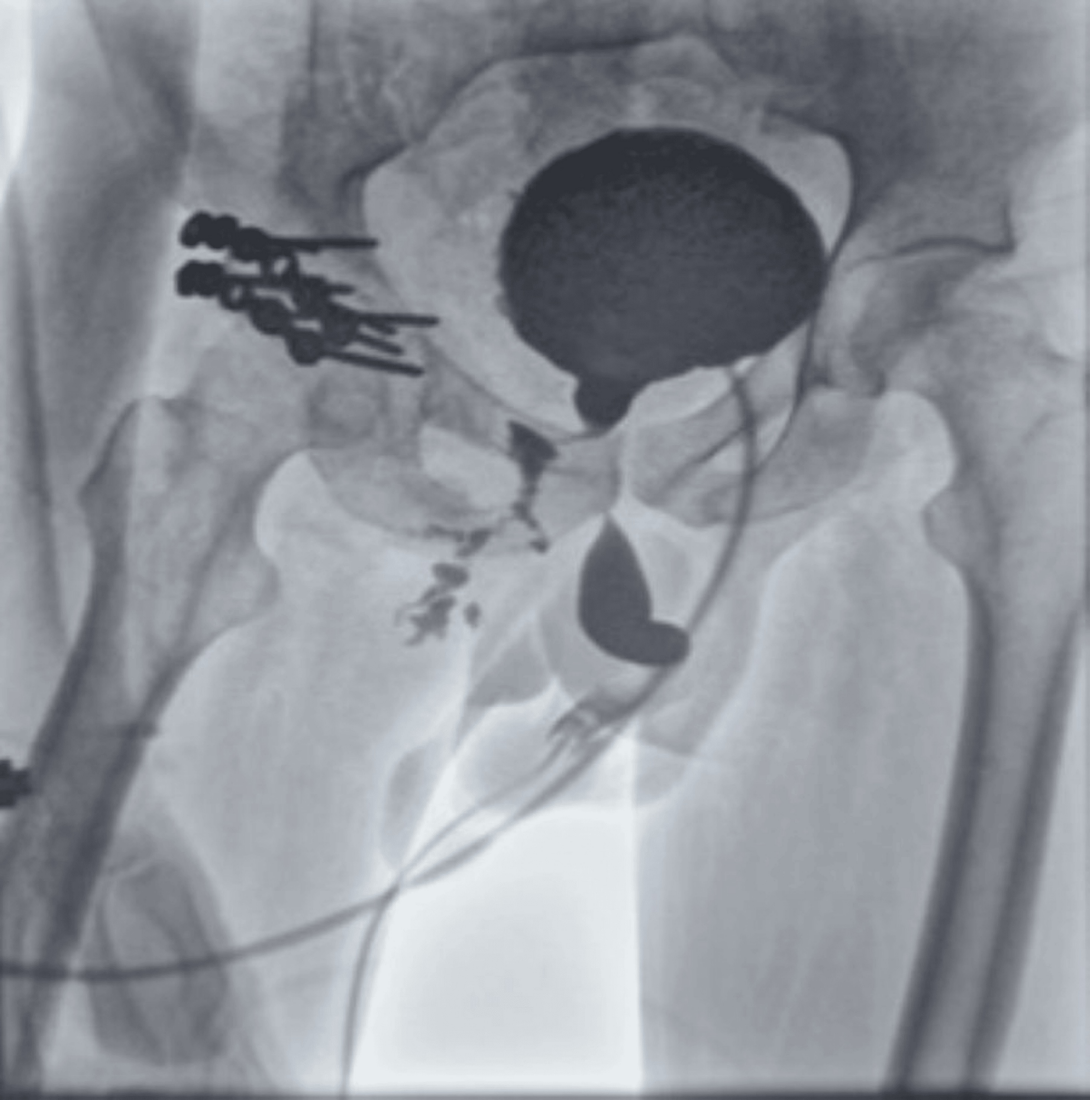
Patient with PFUI. This 41-year-old male patient was initially admitted with fractures of the right pubic ramus and right acetabulum, with accompanying bleeding from the urethral meatus and urinary retention, and suprapubic catheterization was performed due to inability to perform transurethral catheterization. Combined RUG and VCUG provides valuable information regarding the rupture extent and the relations between the distal and proximal urethral stumps and pubic symphysis. In this case, the imaging study revealed inability to advance contrast medium into the bladder and its extravasation, a diagnosis of complete urethral rupture. PFUI: pelvic fracture urethral injury, RUG: retrograde urethrogram, VCUG: voiding cystourethrogram Consent to publish this image was obtained from the patient.

Figure 10 shows pelvic fracture urethral injuries.

## Conclusions

RUG remains the gold standard in the evaluation of urethral strictures and injuries. Nevertheless, VCUG provides essential information that can be missed with RUG alone in specific cases. When used in combination, the two modalities offer a comprehensive evaluation of the urethra. In our practice, we perform VCUG, along with RUG, in every patient that is evaluated for urethral stricture or injury, excluding patients with acute urethral injury. VCUG is easy to perform after the RUG, as it demands only that the bladder be filled with 250-200 cc, and it is valuable in cases of nearly obliterative strictures, fossa navicularis and meatal strictures, posterior urethral stenosis, and pelvic fracture urethral injuries. Also, it provides the added benefit of identification of coexisting bladder pathology and functional abnormalities, which may significantly influence surgical planning. In order to more reliably evaluate and quantify its added clinical benefit, it is essential to conduct studies comparing the urethral lesion site and length calculated intraoperatively with and without the use of VCUG, along with standard RUG.
